# The origin of chromosomal inversions as a source of segmental duplications in the Sophophora subgenus of Drosophila

**DOI:** 10.1038/srep30715

**Published:** 2016-07-29

**Authors:** Eva Puerma, Dorcas J. Orengo, Montserrat Aguadé

**Affiliations:** 1 Departament de Genètica, Microbiologia i Estadística, Facultat de Biologia and Institut de Recerca de la Biodiversitat (IRBio), Universitat de Barcelona, Barcelona, Spain

## Abstract

Chromosomal inversions can contribute to the adaptation of organisms to their environment by capturing particular advantageous allelic combinations of a set of genes included in the inverted fragment and also by advantageous functional changes due to the inversion process itself that might affect not only the expression of flanking genes but also their dose and structure. Of the two mechanisms originating inversions —ectopic recombination, and staggered double-strand breaks and subsequent repair— only the latter confers the inversion the potential to have dosage effects and/or to generate advantageous chimeric genes. In *Drosophila subobscura*, there is ample evidence for the adaptive character of its chromosomal polymorphism, with an important contribution of some warm-climate arrangements such as E_**1+2+9+12**_. Here, we have characterized the breakpoints of inversion E_12_ and established that it originated through the staggered-break mechanism like four of the five inversions of *D. subobscura* previously studied. This mechanism that also predominates in the *D. melanogaster* lineage might be prevalent in the Sophophora subgenus and contribute to the adaptive character of the polymorphic and fixed inversions of its species. Finally, we have shown that the *D. subobscura* inversion breakpoint regions have generally been disrupted by additional structural changes occurred at different time scales.

Structural variation ranging from chromosomal inversions to chromosome fusions does not only lead to genome reorganization through time but it can also contribute to the adaptation of organisms to their environment. In the Drosophila genus where chromosomal polymorphism due to paracentric inversions is widespread, the concordant changes detected between environmental variables and inversion frequencies in multiple species clearly support the adaptive character of this polymorphism. Indeed, parallel latitudinal clines have been detected in two or more continents in species such as *Drosophila melanogaster* and *Drosophila subobscura*, with the frequency of cold-climate arrangements increasing with latitude^1^,^2^,^3^,^4^,^5^. Also, seasonal as well as short- and long-term temporal frequency changes associated to changes in environmental variables have been detected in *D. pseudoobscura* and *D. subobscura*^6^,^7^,^8^, among other species. In those cases, the selective advantage conferred by the successful inversion may be due to its capturing a particular allelic combination of a set of genes included in the inverted fragment, combination that would be preserved through time due to the reduced recombination in inversion heterokaryotypes. It may also be due to the structural change itself since it might affect not only the expression of flanking genes but also their dose and structure (see below).

The identification and molecular characterization of inversion breakpoints has confirmed previous cytological observations indicating that they are not evenly distributed across the genome, with some regions having undergone multiple disruptions both at the short- and long-term time scales^9^,^10^,^11^,^12^. Moreover, the molecular characterization of the breakpoints of diverse polymorphic and rather recently fixed inversions has revealed that inversions can originate either by ectopic recombination between inverted copies of a particular repetitive sequence or by staggered double-strand breaks and subsequent repair^13^. The latter mechanism that generates duplications predominates in the melanogaster group^13^,^14^ and possibly also in the obscura group^11^,^12^. Indeed, 17 of the 29 inversions fixed since the *D. melanogaster*-*D. yakuba* split originated through this mechanism. For polymorphic inversions with breakpoints molecularly characterized, 5 of 8 in *D. melanogaster* and 4 of 5 in *D. subobscura* also originated by this mechanism.

Despite the extensive information supporting the adaptive character of chromosomal polymorphism in different insect and plant species, targets of selection remain largely unknown. Recent genome-wide analyses of variation in *D. melanogaster* have identified some genes within inverted fragments as candidates for having undergone recent adaptive changes^15^,^16^. Also, gene expression analyses of genes flanking inversion breakpoints have revealed in some cases a functional effect of the inversion itself^10^,^17^,^18^. The molecular characterization of polymorphic inversion breakpoints in multiple species becomes an essential task not only to later uncover targets of selection in the inverted region through the footprint that adaptive changes leave on the level and pattern of nucleotide variation but also to assess whether the originating mechanism might have a direct functional effect by changing a gene regulatory environment —if generated by either ectopic recombination or staggered breaks— or an indirect effect through the possible dosage or chimeric effects of duplications —if generated through the staggered breaks mechanism—.

In *D. subobscura*, the analysis of inversions latitudinal clines through time as well as of chromosomal arrangements temporal frequency changes allowed discerning warm-climate from cold-climate arrangements, with the latter increasing with latitude and decreasing with temporal increases in temperature. Among warm-climate arrangements present in Old-World populations of this species, O_3+4+7_ and E_1+2+9+12_ stand out by their contribution to the rapid response to a sudden increase in temperature^19^, whereas arrangements J_1_ and E_1+2+9+12_ exhibit the highest correlation coefficients with changes in latitude^20^.

The E chromosome (Muller’s C element) of *D. subobscura* presents a complex system of chromosomal arrangements that was generated by five mostly overlapping inversions —E_1_, E_2_, E_9_, E_3_ and E_12_ (Fig. 1-A)— that occurred sequentially. The extant chromosomal arrangements E_1+2,_ E_1+2+9_, E_1+2+9+3_ and E_1+2+9+12_are among the most common in the Mediterranean area^21^,^22^,^23^. We have so far characterized the breakpoints of four of these inversions: E_1_, E_2_ and E_9_, leading from the ancestral cold-climate E_st_ arrangement to the warm-climate E_1+2+9_ arrangement, and E_3_ that like inversion E_12_ originated on the E_1+2+9_ arrangement (Fig. 1-A^11^,^12^). Here, we have identified and characterized the breakpoints of the last of these inversions — inversion E_12_—, which will not only extend our knowledge on inversion generating mechanisms in the highly polymorphic species *D. subobscura* but most importantly it will facilitate uncovering in the near future what renders each of the chromosomal arrangements of the E_1+2_ complex adaptive. Moreover, we have characterized the regions flanking inversion E_12_ breakpoints through the Drosophila phylogeny to better understand the contribution of fragile sites to chromosomal evolution.

## Results

### E_12_ inversion breakpoints

**Figure 1:**
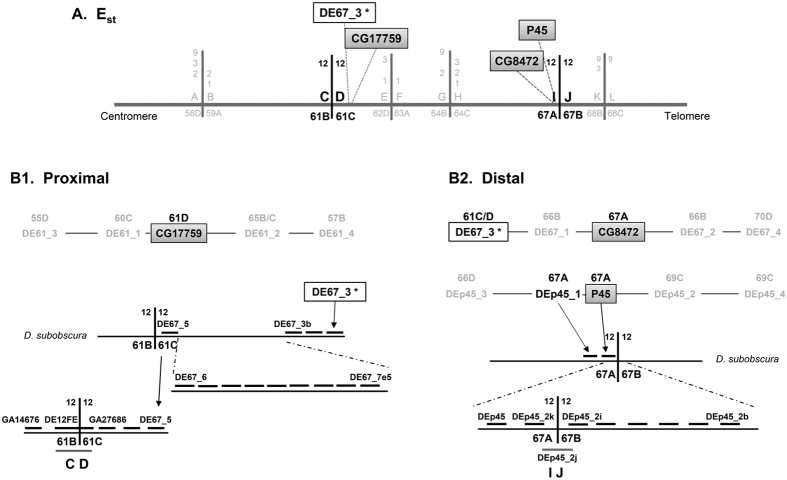
Chomosome walking strategy. (**A**) Schematic representation of the E chromosome standard arrangement of *D. subobscura.* Continuous vertical lines represent the different inversion breakpoints −E_1_, E_2_, E_3_, E_9_ and E_12_− that are labeled consecutively with pairs of capital letters (*e.g.,* AB, CD, EF, GH, etc.) from the most proximal to the most distal breakpoint. The breakpoints involved in the *D. subobscura* E_12_ inversion are highlighted in large bold letters. Numbers on both sides of each continuous vertical line refer to the inversions delimited by each breakpoint, whereas its location (section) on the Kunze-Mühl and Müller^24^ map is indicated below the breakpoint name (see text for breakpoint CD). The three initial markers used to initiate chromosome walks to identify the breakpoints of inversion E_12_ in standard chromosomes (CD and IJ) are included in grey-shaded boxes whereas a new marker derived from the IJ chromosomal walk —DE67_3*— is included in a clear box. (**B**) Simplified scheme of the chromosome walks (B1 and B2) performed to identify the proximal −CD− and distal −IJ− breakpoint regions (not at scale) of inversion E_12_. Markers used to initiate each chromosome walk are highlighted as in section A of this figure. In each scheme, only the most relevant probes names are indicated. The location of probes on the Kunze-Mühl and Müller^24^ map of *D. subobscura* is indicated with the section number and letter. In the three initial chromosomal walks, probes moving away from the breakpoint regions are depicted in small size font. In the subsequent chromosome walks (see Supplementary Figs S2 and S4, for details), probes are presented above a line representing the *D. subobscura* chromosome. In each breakpoint region, a thick vertical line represents the breakpoint itself and the probe spanning the breakpoint is represented by a thick grey line.

We have molecularly characterized by chromosome walking the E_12_ inversion breakpoints in the E_st_ arrangement (CD and IJ; Fig. 1-A and Supplementary Fig. S1) as well as in the E_1+2+9+12_arrangement (JD and IC; Supplementary Fig. S1). At the cytological level and according to the Kunze-Mühl and Müller^24^ map for the E_st_ arrangement, the E_12_ inversion breakpoints would be located at sections 61C/61D and 67A/67B for the proximal and distal breakpoint, respectively. However, a later comparison of the banding pattern of chromosomal arrangements E_st_ and E_1+2+9+12_ led Cuenca *et al.*^25^ to propose section 61B/61C as the proximal breakpoint.

**Figure 2:**
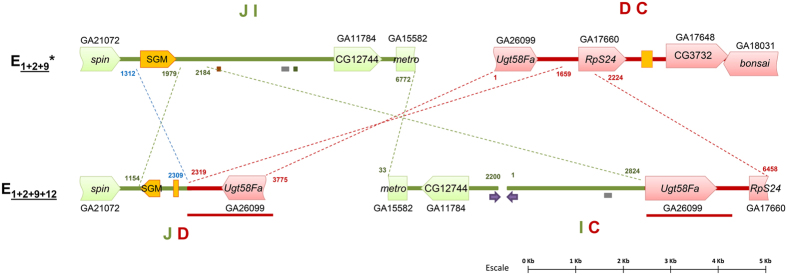
Schematic representation of inversion E_12_ breakpoint regions in chromosomal arrangements E_1+2+9_ and E_1+2+9+12_ . Breakpoint regions in E_1+2+9_ are color-coded and annotated as in Supplementary Fig. S1. Within each sequenced fragment, coding regions and transposable element SGM are represented by large boxes and intergenic regions by thick lines, whereas other transposable elements and long inverted repeats are represented by small boxes and arrows, respectively, below each breakpoint region. Thin discontinuous lines between arrangements indicate the limits and orientation of homologous regions, with numbers indicating their location in the sequenced fragments. The thick red line below the IC and JD breakpoints indicates the region that was duplicated during the inversion process.

Three molecular markers previously located near E_12_ inversion breakpoints were used as starting points for the corresponding chromosomal walks. The CG17759 marker previously located at section 61D was the starting point to identify the proximal CD breakpoint, whereas the P45 and CG8472 markers located at section 67A were the starting points to identify the distal IJ breakpoint. In a first approximation, two probes were designed upstream and downstream from each marker at distances ranging from 5 kb to 80 kb following the *D. pseudoobscura* genome. In this initial effort, only one of the 12 probes designed −DEp45_1− gave a hybridization signal near the corresponding breakpoint —the distal IJ breakpoint— (Fig. 1-B and Supplementary Fig. S2). As probe DEp45_1 was located at section 67A, but seemed more distant to the breakpoint than probe P45, several consecutive probes were designed between the P45 and DEp45_2 markers that covered the entire region despite the location of the latter marker at section 69C in *D. subobscura* (Supplementary Fig. S2). Probes DEp45_2k, DEp45_2j and DEp45_2i gave single strong signals at sections 67A, 67A/67B and 67B, respectively, on E_st_ chromosomes. Given the breakpoint location at section 67A/67B, these results would indicate that probe DEp45_2j included the breakpoint. Hybridization of this probe on E_1+2+9+12_ chromosomes gave two strong signals corresponding to the JD and IC breakpoints location, which confirmed that it spanned the IJ breakpoint (Fig. 1-B2, and Supplementary Figs S2 and S3). It should be added that this probe also gave multiple weak signals mainly at centromeric and telomeric regions, probably due to it containing some repetitive sequences. The DEp45_2j fragment was completely sequenced upon its amplification in the *ch cu* strain. The sequenced fragment (~6.8-kb long) contains a partial orthologous region of the *spin* gene, a nearly complete SGM transposable element^26^, the CG12744 gene ortholog, and a partial orthologous region of the *metro* gene (Fig. 2).

The failed attempt to use probes designed around the CG17759 marker to walk towards the CD breakpoint (Fig. 1-B1 and Supplementary Fig. S2), and the serendipitous localization of probe DE67_3 at section 61C/61D (Fig. 1-B2) led us to use the latter probe as the starting point to identify the CD breakpoint (Fig. 1-B1 and Supplementary Fig. S4). Since the nearest probe to DE67_3 according to the *D. pseudoobscura* genome (DE67_1) did not hybridize at the CD breakpoint region (Fig. 1 and Supplementary Fig. S4), two additional sets of probes were designed in the opposite direction following the *D. pseudoobscura* genome. A first set of probes (DE67_3a to DE67_7e) hybridized either at section 61C/61D (like probe DE67_3) or at section 61C (i.e., moving closer to the CD breakpoint). A second set (DE67_8 to DE67_10) hybridized at a different chromosomal region (Supplementary Fig. S4). Since colinearity between *D. pseudoobscura* and *D. subobscura* had been lost, the chromosome walk was continued using the *D. melanogaster* genome. Four of the five newly designed probes —DE67_7e1 to DE67_7e5— hybridized at the CD breakpoint region but not in the expected direction, whereas the fifth one did at a different region. The loss of colinearity of this region with both the *D. pseudoobscura* and *D. melanogaster* genome sequences prevented to further move towards the CD breakpoint using these reference genomes. However, the later availability of an improved version of *D. subobscura* genome (draft2; BSI, Barcelona Subobscura Initiative) with an ~40 kb scaffold that included the DE67_5 orthologous region and other *D. pseudoobscura* orthologous regions not previously explored (Supplementary Fig. S4), allowed designing new probes that finally led to the identification of the breakpoint. Indeed, probe DE12EF gave a strong signal on E_st_ chromosomes at section 61B/61C (CD breakpoint) and two signals on E_1+2+9+12_ chromosomes at sections 61C next to 67B (JD breakpoint) and 61B next to 67A (IC breakpoint), respectively, corroborating that this probe spanned the CD breakpoint (Supplementary Fig. S3). Probe DE12EF was subsequently amplified and completely sequenced in the *ch cu* strain. The sequenced fragment (~6-kb long) contains orthologous regions corresponding to genes *Ugt58Fa*, *RpS24* and CG3732, and a partial *bonsai* gene, as well as remains of an SGM element (Fig. 2).

Upon identification of the CD and IJ breakpoint regions in standard chromosomes, fragments spanning the JD and IC breakpoint regions in E_1+2+9+12_ chromosomes were amplified with the corresponding combination of oligonucleotides (Supplementary Fig. S1). *In situ* hybridization of fragments amplified on E_1+2+9+12_ chromosomes gave two signals on E_st_ chromosomes (Supplementary Fig. S5). These results confirmed that the amplified fragments include the JD and IC breakpoints. However, these fragments also gave two signals on E_1+2+9+12_ chromosomes (see below).

The JD fragment (~3.8-kb long) was completely sequenced in the OF19 (E_1+2+9+12_) strain whereas the IC fragment (~8.6-kb long) was almost completely sequenced in this strain given the presence of at least two ~400 nt-long identical inverted sequences that interfered with sequencing.

The detailed analysis of all sequenced fragments through pairwise comparison allowed delimiting the breakpoints and also their molecular characterization (Fig. 2). The IJ and JD sequence comparison allowed us to determine that the distal breakpoint of inversion E_12_ occurred at the intergenic region between the *spin* and CG12744 genes and more specifically beyond the SGM element present at the JI breakpoint. However, the element present at the JD region is degraded and in the inverted sense, which suggests a microinversion that included the SGM element followed by its degradation. The CD and IC sequence comparison allowed delimiting the distal breakpoint of inversion E_12_ to the intergenic region between genes *Ugt58Fa* and *RpS24*. The presence at the IC breakpoint of a complete copy of the *Ugt58Fa* gene and of part of both its flanking regions indicates that this fragment was duplicated as a result of the chromosomal inversion that originated through the staggered double-strand break mechanism. This duplication explains the double signal observed at the breakpoint regions in the E_st_ and E_1+2+9+12_ chromosomal arrangements both when using the JD and IC fragments as probes (Supplementary Fig. S5).

### E_12_ breakpoint regions across the Drosophila Genus

The chromosome walks performed to identify the E_12_ breakpoints revealed a high frequency of colinearity disruptions among the genomes of *D. subobscura, D. pseudoobscura* and *D. melanogaster* (Supplementary Figs S2 and S4). Additionally, during the annotation of the regions spanning inversion E_12_ breakpoints through their comparison to the *D. pseudoobscura* and *D. melanogaster* genomes, we noticed that one of the genes flanking the CD breakpoint —*Ugt58Fa*— is located in *D. pseudoobscura* in a different chromosomal element. These observations motivated us to characterize both E_12_ breakpoint regions across the Drosophila phylogeny^27^,^28^, and more specifically, the two genes adjacent to each breakpoint as well as their neighbouring genes.

#### CD breakpoint region

Supplementary Fig. S6 summarizes the comparative analysis performed concerning colinearity breaks across the Drosophila phylogeny of the two genes flanking the CD breakpoint in the E_st_ arrangement −*Ugt58Fa* and *RpS24*−. The block formed by these genes and their immediate neighbours in the E_st_ arrangement −*CG3732-RpS24-Ugt58Fa-CG2852*− is conserved in all species considered except among the members of the obscura group. This 4-genes block would therefore predate the diversification of the Drosophila genus. In the obscura group, this order would have been maintained in chromosomal arrangements of *D. subobscura* lacking the E_12_ inversion (*e.g*., E_st_, E_1+2_, E_1+2+9_ and E_1+2+9+3_). In addition, the *Ugt58Fa* gene would have been transposed from Muller’s element C to element B in the ancestor of *D. pseudoobscura* and *D. persimilis*. Concerning the two genes flanking the proximal E_12_ inversion breakpoint, a total of three disruptions would have occurred across the Drosophila phylogeny —two at the 3′ downstream region of the *Ugt58Fa* gene and one at its 5′ upstream region— as a result of one interchromosomal transposition that became fixed in the ancestor of *D. pseudoobscura* and *D. persimilis*, and of one paracentric inversion that segregates in *D. subobscura* as part of the E_1+2+9+12_ arrangement.

#### IJ breakpoint region

The comparative analysis performed concerning colinearity breaks across the Drosophila phylogeny of the two genes flanking the IJ breakpoint in the E_st_ arrangement −*spin* and *CG12744*− revealed a more complex evolutionary history (Supplementary Fig. S7). The presence of the CG30095-*spin*-CG18446-CG12744-*Sec24AB* block in species of the Drosophila subgenus and in *D. willistoni*, and the presence of two different subsets of these genes in species of the melanogaster subgroup —CG30095-*spin*-CG18446 in *D. ananassae*, and CG18446-CG12744-*Sec24AB* in the other five species— clearly support the ancestral character of this 5-genes block. Concerning the two genes flanking the distal E_12_ inversion breakpoint, the CG12744 gene might have been lost independently in *D. ananassae* and *D. grimshawi* (Supplementary Fig. S7). Additionally, the intergenic region separating the *spin* and CG12744 genes would have been disrupted twice across the Drosophila phylogeny as a result of one paracentric inversion (E_12_) that segregates in *D. subobscura* as part of the E_1+2+9+12_ arrangement, and the loss of gene CG18446 in the ancestor of the obscura group. At least an additional disruption would have occurred at the *spin* downstream region as a result of gene insertions in the *D. grimshawi* lineage.

## Discussion

Structural variation due to chromosomal inversions was extensively studied during the last century in multiple species of Drosophila and other diptera with polytene chromosomes. Despite the extensive evidence accumulated in this period for the adaptive character of chromosomal inversion polymorphism in diverse species of Drosophila, such as *D. subobscura*, *D. pseudoobscura* and *D. melanogaster*, only in the post-genomic era have we witnessed a renewed and generalized interest for chromosomal inversion polymorphism^29^. This renewed interest emerged from the possibility to generate genome-wide population datasets that would allow detecting putative targets of selection across the genome, and more specifically along the inverted fragment. There is still an enormous gap between the progress that can be achieved in different species concerning chromosomal polymorphism given the different availability and generally very differential quality of their genome sequences, if any. Indeed, model species such as *D. melanogaster* have a good reference genome, whereas in other species multiple non-ordered scaffolds might only partially cover each chromosomal element.

In the Drosophila genus, most of the breakpoints of polymorphic inversions so far identified and characterized have required laborious chromosome walks^10^,^11^,^12^,^17^,^30^,^31^,^32^,^33^,^34^,^35^. The availability of complete genome sequences has so far facilitated the identification of one of the multiple inversions that segregate in *D. pseudoobscura* —the *Arrowhead* inversion^36^— whereas the breakpoints of several inversions segregating in the model species *D. melanogaster* —with one of the most complete assembled genomes— have been identified from genome-wide population datasets^14^.

Our characterization of the breakpoints of inversion E_12_ has revealed that this inversion of *D. subobscura* originated through the staggered-breaks mechanism, similarly to polymorphic inversions O_3_, E_1_, E_9_ and E_3_ and unlike inversion E_2_^11^,^12^,^35^. This finding corroborates that the predominant mechanism that originates inversions in *D. subobscura* is the staggered-breaks mechanism as it also is in *D. melanogaster* where information has been obtained for both polymorphic inversions and recently fixed inversions. In contrast, inversions seem to have been mostly generated through ectopic recombination between repetitive elements in the repleta group^10^,^31^,^32^,^33^,^34^. Even if the number of inversions with breakpoints molecularly characterized is scarce in polymorphic Drosophila species, present data suggest that the staggered-breaks mechanism might be characteristic of the Sophophora subgenus.

Polymorphic inversions are those that have attained a certain frequency in the population due to the action of either drift —if they do not affect the fitness of their bearers— or positive selection —if they increase their fitness. In Drosophila, there is ample evidence for inversion polymorphism being adaptive, which would imply the action of positive selection not only for the initial increase in frequency of the corresponding inversions but also for their later maintenance in the population. It is easy to envisage that inversions that disrupt essential coding regions or separate them from their regulatory region would have a detrimental effect on fitness and would be rapidly lost from the population. However, it is also easy to envisage that in those lineages where the staggered-breaks mechanism is the prevalent mechanism, it is more unlikely that inversions would have negative effects when they occur given that a functional copy of the affected gene/s would be present at least at one of the breakpoint regions. Moreover, the duplicated fragments would confer to the new inversion the potential to have positive effects even though this potential would vary depending on their extent and content. In *D. subobscura* as is also the case in *D. melanogaster*, duplicated fragments vary in size between a few hundred base pairs —in inversions O_3_ and E_1_— to 8 Kb —in inversion E_9_^11^,^12^,^35^. Except for the shortest fragments, duplications include one or more complete or partial genes. Although the duplication of complete genes has the potential to have dosage effects, our analysis of duplications of partial genes has not revealed in any case the putative generation of new transcription units and/or of chimeric genes (results not shown). It should be noted that in other Drosophila species, some functional effects have been experimentally detected as is the case at the distal breakpoint of the *D. melanogaster* In(3L)Payne inversion —with three disrupted transcripts in flies homozygous for the inversion^17^— and also at the proximal breakpoint of the 2j inversion of *D. buzzatii* —where a flanking gene was nearly silenced by an antisense RNA that originated in a transposable element inserted at the breakpoint^18^.

In the Drosophila genus, cytological studies of inversion polymorphism had revealed that inversions are not evenly distributed either among species or among and within chromosomal elements of polymorphic species^37^. In this genus, the comparative analysis of multiple genomes across the phylogeny also revealed the uneven distribution of inversion breakpoints across the genome^9^,^38^. Our previous characterization of the breakpoints of five inversions that segregate in *D. subobscura* revealed i) that breakpoints with cytological evidence for breakpoint reuse (inversions E_1_, E_2_, E_9_ and E_3_) had been in most cases also reused at the molecular level; ii) that some inversion breakpoint regions had been affected in the same species by other structural rearrangements prior or after the primary inversion (inversions E_1_ and E_2_); and iii) that some inversion breakpoint regions (inversions O_3_, E_1_, E_2_, E_9_ and E_3_) had been affected by other structural rearrangements across the Drosophila phylogeny^11^,^12^,^35^. The characterization of the E_12_ inversion breakpoints does not only provide further evidence of the fragility of inversion breakpoints —with a microinversion in the distal breakpoint of the inverted chromosome— but it also supports the high general fragility of this chromosomal element as evidenced by the multiple colinearity breaks detected during both chromosomal walks performed to identify inversion E_12_ breakpoints (Supplementary Figs S2 and S4). Moreover, several disruptions have affected the breakpoints flanking genes through the Drosophila phylogeny with a major incidence in the obscura group of species (Supplementary Fig. S6).

In summary, we have completed the characterization of the breakpoints of five chromosomal inversions involved in a complex inversion system of *D. subobscura*, which arrangements can generally be considered warm-climate arrangements and are among the most frequent in the Mediterranean area. We have shown that most of these inversions originated by the staggered-break mechanism, a mechanism that by generating duplications confers the inversion the potential to have positive effects on the fitness of its bearers. Finally, our characterization of inversion breakpoint regions in *D. subobscura* and across the Drosophila phylogeny has revealed their breakage-prone nature since multiple structural changes have generally affected these regions.

## Materials and Methods

The *ch cu* and OF19 strains of *D. subobscura* that are homokaryotypic for the E_st_ and E_1+2+9+12_ chromosomal arrangements, respectively, were used to molecularly identify the breakpoints of inversion E_12_ and to subsequently sequence the breakpoint regions. The OF19 strain was obtained through over 13 generations of sibmating from isofemale lines established upon collection in Observatori Fabra (Barcelona, Catalonia, Spain), as reported in Puerma *et al.*^11^.

Molecular markers previously located near the inversion breakpoints were used as starting points for breakpoints identification in E_st_ and E_1+2+9+12_ chromosomes (Fig. 1). The CG17759 (*Galpha49B*) marker previously located at section 61D of the E chromosome^39^ was used as starting point to identify the proximal breakpoint −CD− according to an E_st_ arrangement. Markers P45 and CG8472 (*Cam*) previously located at section 67A of the E chromosome^39^,^40^ were used as starting points to identify the distal breakpoint −IJ− relative to an E_st_ arrangement. P45 refers to a recombinant λ bacteriophage clone randomly isolated from a *D. subobscura* genomic library, which insert content was characterized by phage DNA purification with the Qiagen Lambda Mini Kit, digestion with *Eco*RI and cloning of the digestion product into the *pBluescript II SK* (Stratagene) vector. Subclones with inserts were identified through PCR amplification with the T3 and T7 universal primers and subsequently sequencing the ends of the different subcloned fragments. The *discontiguous megablast* algorithm (http://flybase.org/blast/) was used to find and delimit the orthologous regions of the P45 phage insert in the *D. pseudoobscura* and *D. melanogaste*r genomes.

E_12_ inversion breakpoints were identified through chromosome walks using the *D. pseudoobscura* and *D. melanogaster* genome sequences, as well as some scaffolds from the *D. subobscura* genome sequence (Barcelona Subobscura Initiative [BSI]). For a detailed description of the chromosome walking strategy see Puerma *et al.*^11^. Probes were amplified by PCR using genomic DNA from the *ch cu* strain, biotin labeled and *in situ* hybridized on polytene chromosomes of the *ch cu* and OF19 *D. subobscura* strains. Oligonucleotides for probes amplification were designed directly on *D. subobscura* sequences. Hybridization signals allowed walking towards each breakpoint and to eventually cross it. All steps of the *in situ* hybridization procedure were performed as described in Montgomery *et al.*^41^ and hybridization signals were located on the cytological map of *D. subobscura*^24^. Digital images at a 400 magnification were obtained using a phase contrast Axioskop 2 Zeiss microscope and a Leica DFC290 camera.

To sequence the CD and IJ breakpoint regions in E_st_ chromosomes, and the JD and IC breakpoint regions in E_1+2+9+12_ chromosomes, the fragment spanning each breakpoint was PCR amplified using TaKaRa DNA polymerase (Takara Bio Inc) and oligonucleotides anchored at its flanking regions (Supplementary Fig. S1). The amplified fragments were sequenced using primer walking whenever necessary. MultiScreen PCR plates (Millipore) were used to purify amplicons prior to their sequencing with the ABI PRISM version 3.2 cycle sequencing kit, with sequencing products separated on an ABI PRISM 3730 sequencer. All sequences were obtained on both strands and assembled using the DNASTAR package^42^. When sequences could not be obtained directly from PCR products, we used the cloning and sequencing strategy described in Puerma *et al.*^11^. Sequences newly obtained have been deposited in the EMBL/GenBank Data Libraries under accession numbers LT598605 to LT598609.

### Sequence analysis

All breakpoint regions were annotated with genes by comparison with the *D. pseudoobscura* genome of FlyBase (http://flybase.org/) using BLAST tools and analyzed to detect repeated motifs using RepeatMasker. The newly sequenced breakpoint regions were compared among them using the Align Sequences Nucleotide BLAST utility at NCBI webpage in order to finely establish each breakpoint and to determine putative duplications resulting from the inversion process. BLAST tools were also used to identify homologs of the genes flanking each breakpoint and their neighbors in the first 12 sequenced genomes of the Drosophila genus^27^.

## Additional Information

**Accession codes:** LT598605 to LT598609.

**How to cite this article**: Puerma, E. *et al.* The origin of chromosomal inversions as a source of segmental duplications in the Sophophora subgenus of Drosophila. *Sci. Rep.* **6**, 30715; doi: 10.1038/srep30715 (2016).

## Supplementary Material

Supplementary Information
