# Supplementary material for: The origin of chromosomal inversions as a source of segmental duplications in the Sophophora subgenus of Drosophila
Source: Sci Rep. 2016 Jul 29;6:30715. doi: 10.1038/srep30715 (PMC4965816; doi:10.1038/srep30715)
Supplement: Supplementary Information [file srep30715-s1.pdf]

Supplementary information

The origin of chromosomal inversions as a source of segmental duplications in the Sophophora subgenus of Drosophila

Eva Puerma, Dorcas J. Orengo, Montserrat Aguadé

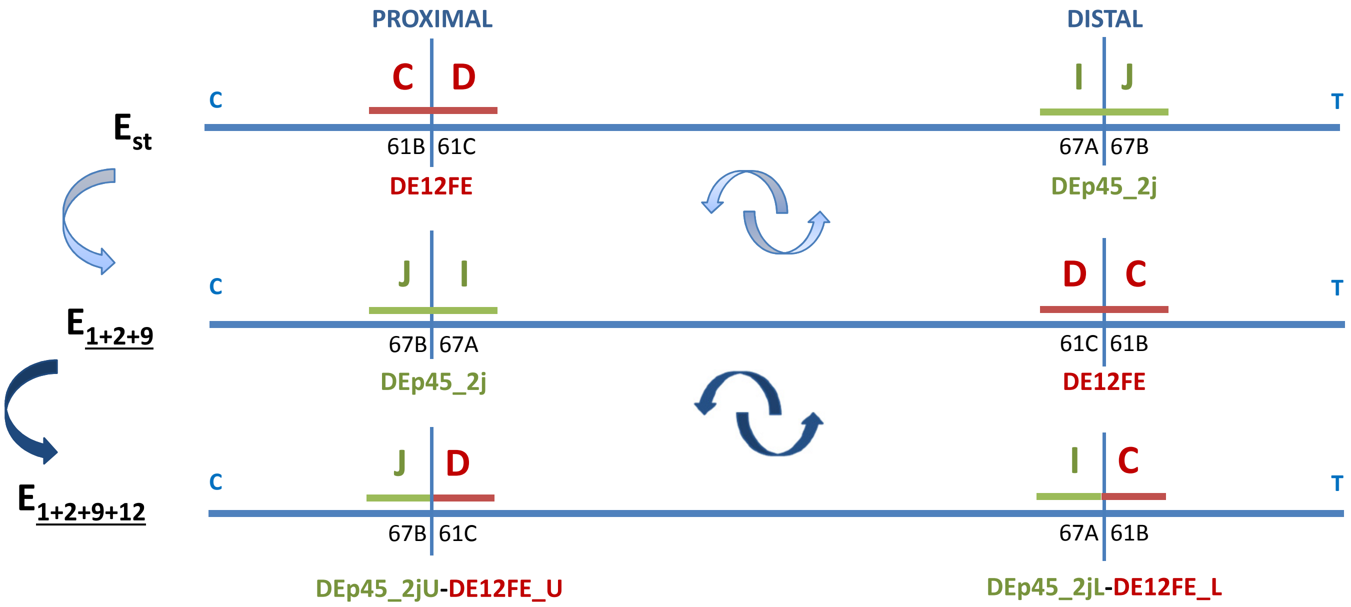

Supplementary Figure S1. Schematic representation of E_{12} breakpoints in chromosomal arrangements E_{st}, $E_{\underline{1+2+9}}$ and $E_{\underline{1+2+9+12}}$. Horizontal blue lines represent that part of the E chromosome affected by inversion E_{12}, whereas the vertical lines represent the inversion breakpoints. The terms proximal and distal refer to the location of inversion E_{12} breakpoints in the E_{st} arrangement using the same notation than in Figure 1. Double arrows highlight inversion events between the three arrangements whereas arrows on the left side of the image represent the sequential accumulation of inversions (light blue, of inversions E_1, E_2 and E_9; dark blue, inversion E_{12}). Short horizontal lines represent the regions spanning each breakpoint in arrangements E_{st} (red, proximal; green, distal), $E_{\underline{1+2+9}}$ and in arrangement $E_{\underline{1+2+9+12}}$. T, telomere; C, centromere.

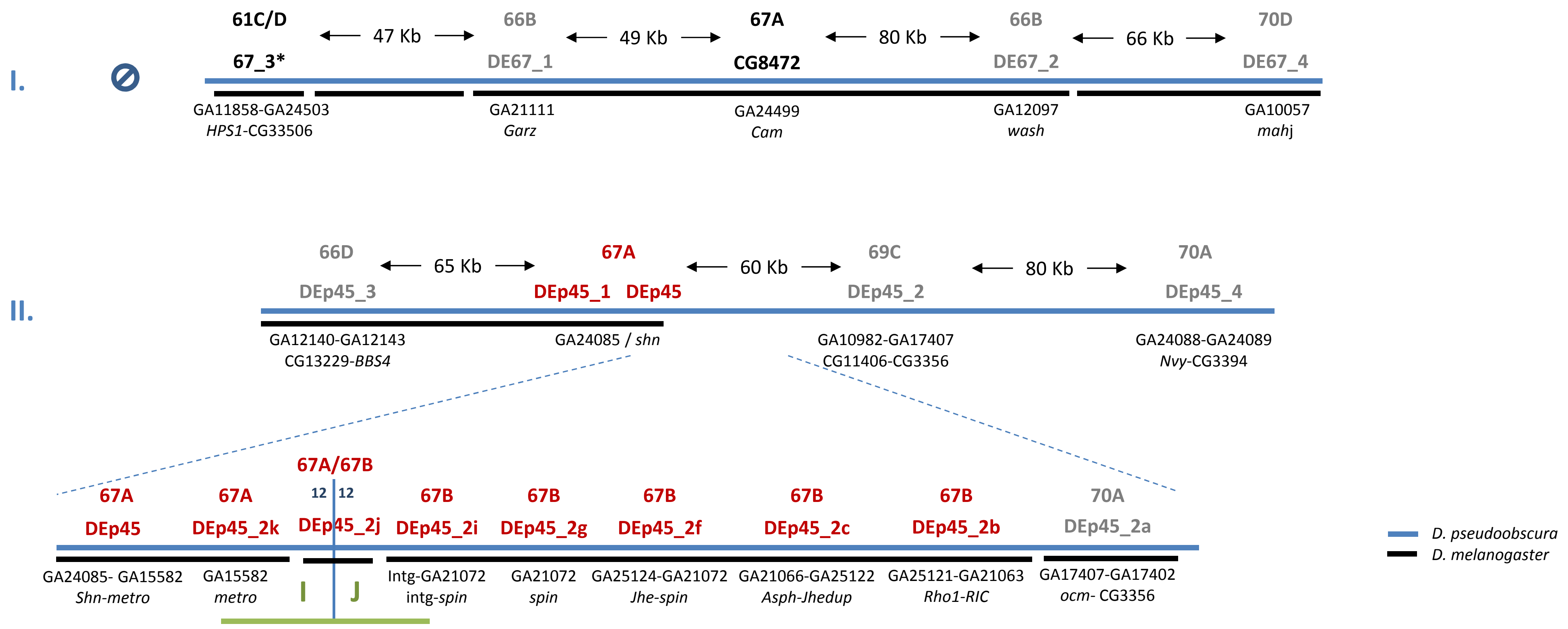

Supplementary Figure S2. Distal (IJ) breakpoint chromosome walk. Detailed schematic representation of the chromosome walk performed to identify the IJ breakpoint (not at scale). Blue horizontal lines represent part of the *D. pseudoobscura* chromosome, whereas black horizontal lines represent the corresponding syntenic blocks in *D. melanogaster*. Probe names and their location (section) on the Kunze-Mühl and Müller (1958) map of *D. subobscura* are indicated above those lines. Red-colored probes are those leading to the successful identification of the IJ breakpoint. A blue vertical line represents the IJ breakpoint and the probe spanning the breakpoint is represented by a thick green line. A crossed blue circle represents an unsuccessful chromosome walk. An asterisk indicates that the labeled probe was used to identify the CD breakpoint.

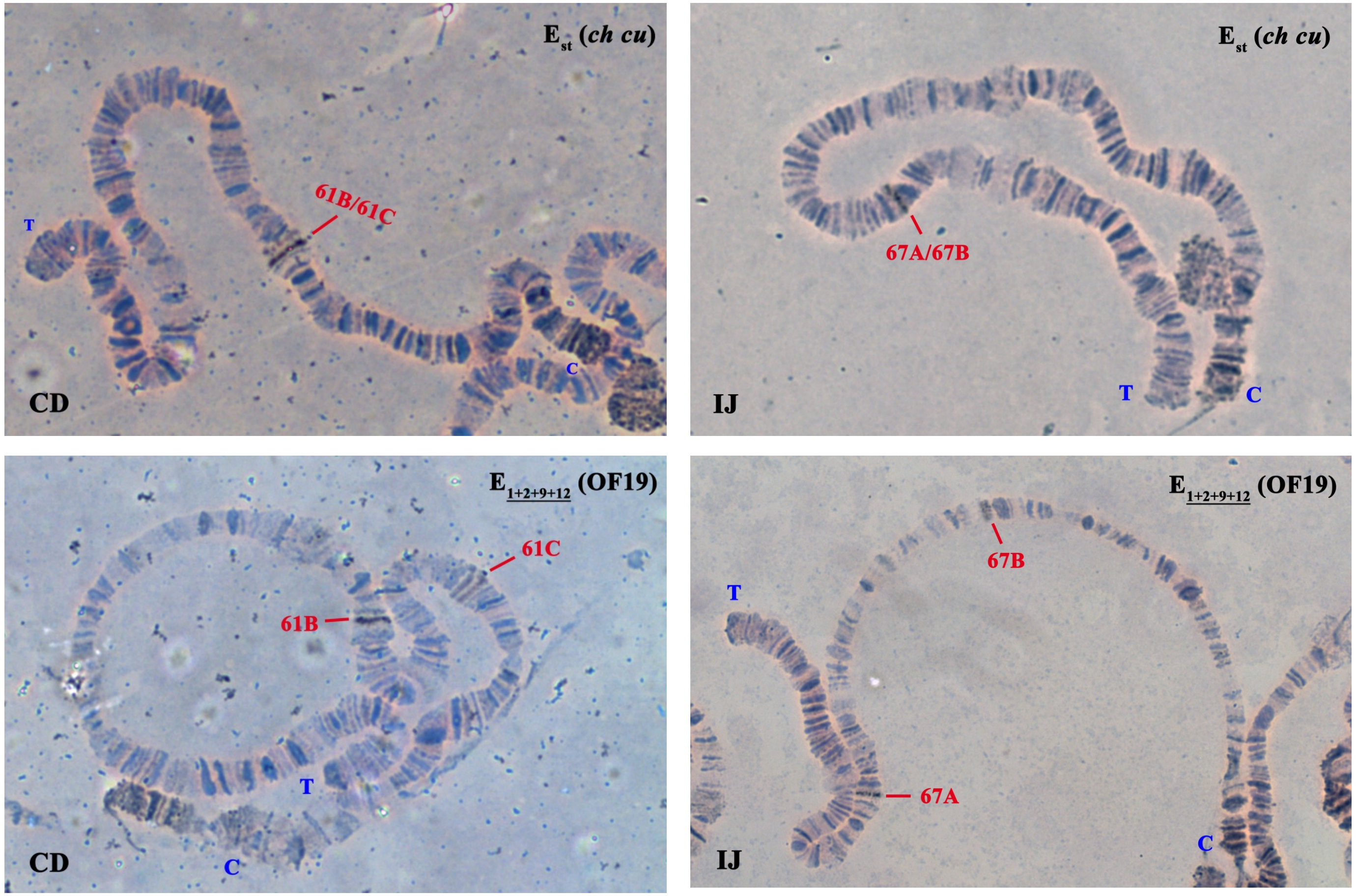

Supplementary Figure S3. ***In situ*** **hybridization results using probes spanning the E_{12} inversion breakpoints in the *ch cu* strain.** Results of the *in situ* hybridizations performed on the *ch cu* (upper part) and OF19 (lower part) strains using fragments CD and IJ that span inversion E_{12} breakpoints in the *ch cu* strain. Hybridization signals are marked with a red line and their cytological location is also indicated in red. T, telomere; C, centromere.

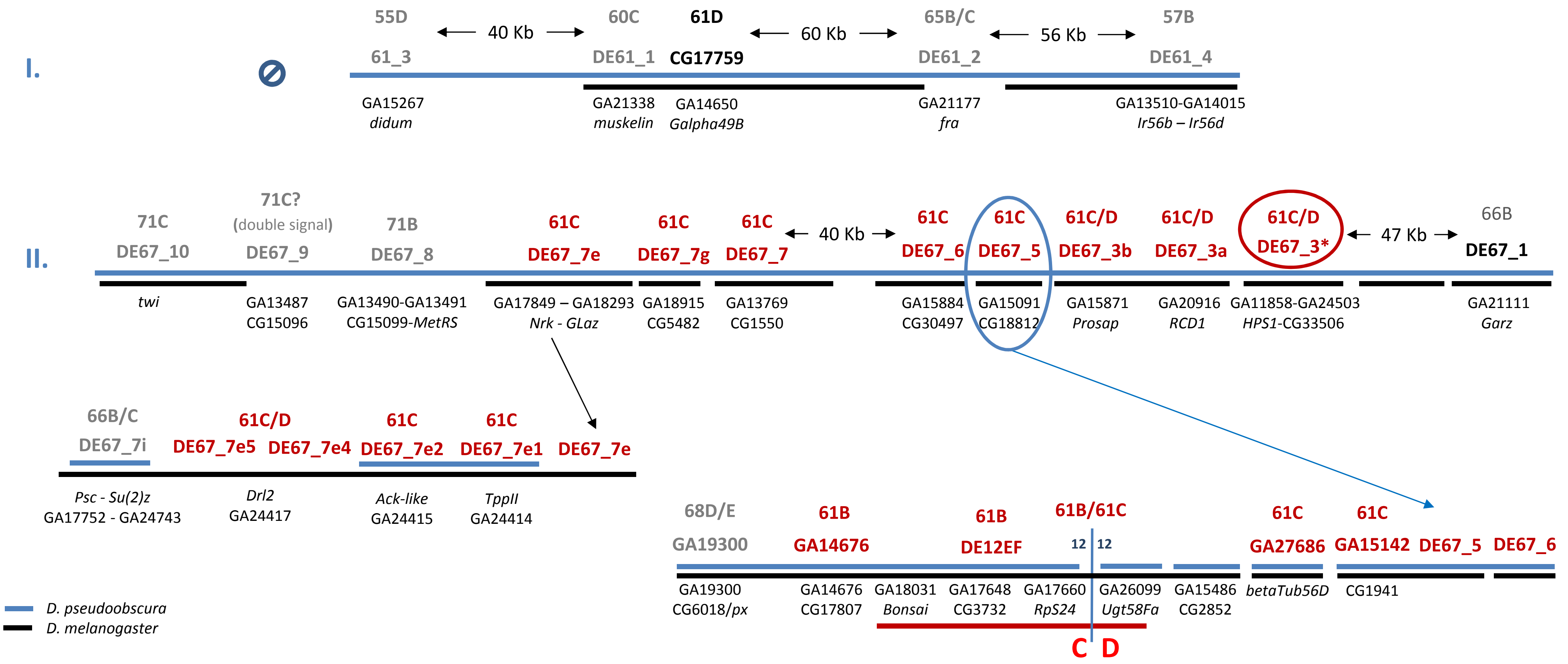

Supplementary Figure S4. Proximal (CD) breakpoint chromosome walk. Detailed schematic representation of the chromosome walk performed to identify the CD breakpoint (not at scale). Blue horizontal lines represent the *D. pseudoobscura* chromosome, whereas black horizontal lines represent the corresponding syntenic blocks in *D. melanogaster*. Probe names and their location (section) on the Kunze-Mühl and Müller (1958) map of *D. subobscura* are indicated above those lines. Red-colored probes are those leading to the successful identification of the CD breakpoint, with that used to initiate each walk within an oval. A blue vertical line represents the CD breakpoint and the probe spanning the breakpoint is represented by a thick red line. A crossed blue circle represents an unsuccessful chromosome walk. An asterisk indicates that the labeled probe was obtained during the unsuccessful chromosome walk initiated to identify the IJ breakpoint (Fig. 1 and Supp. Fig. S2).

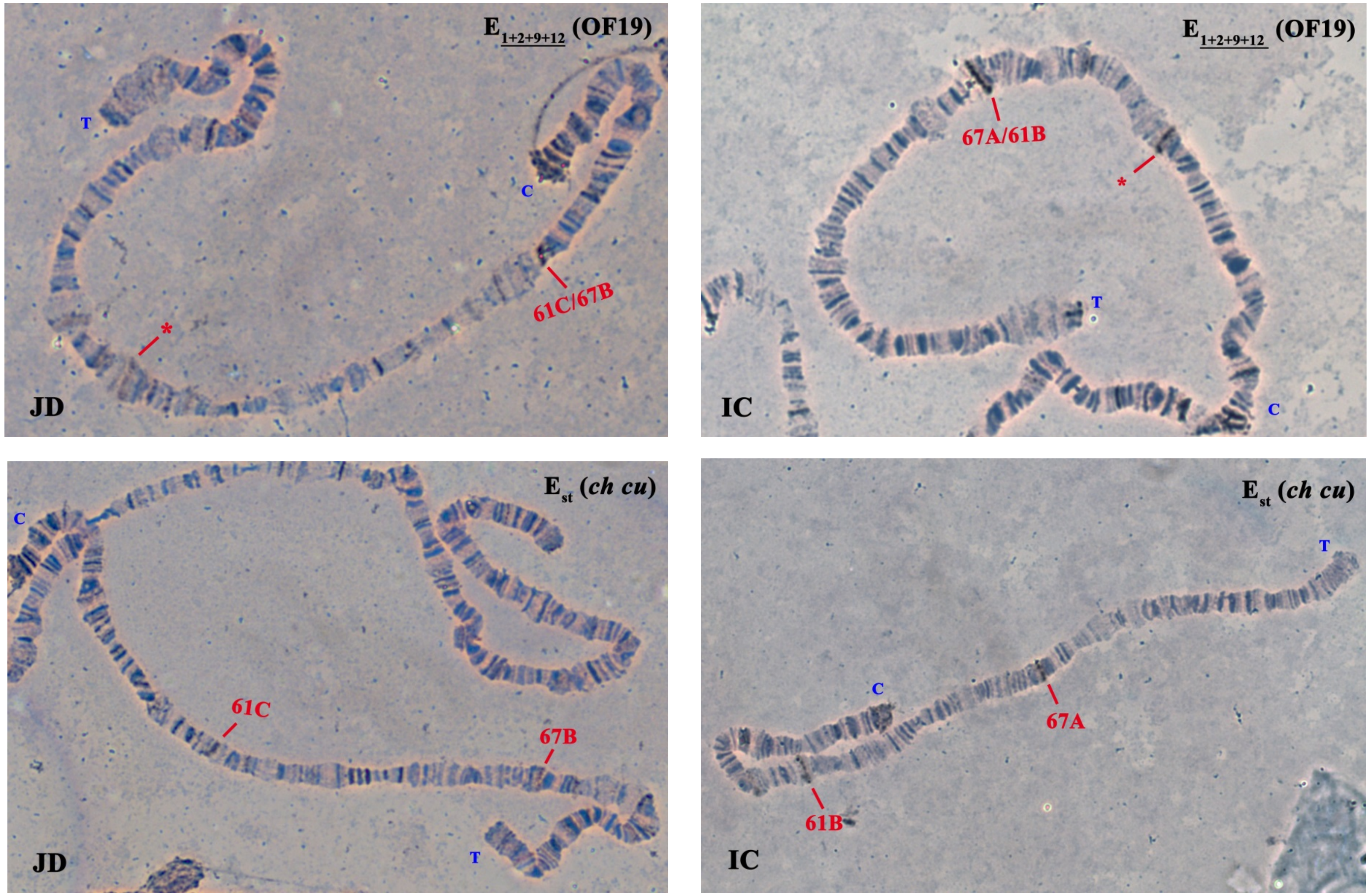

Supplementary Figure S5. ***In situ*** **hybridization results using probes spanning the E_{12} inversion breakpoints in the OF19 strain.** Results of the *in situ* hybridizations performed on the *ch cu* (upper part) and OF19 (lower part) strains using fragments JD and IC that span inversion E_{12} breakpoints in the OF19 strain. Hybridization signals are marked with a red line (and the duplication breakpoint region with an asterisk) and their cytological location is also indicated in red. T, telomere; C, centromere.

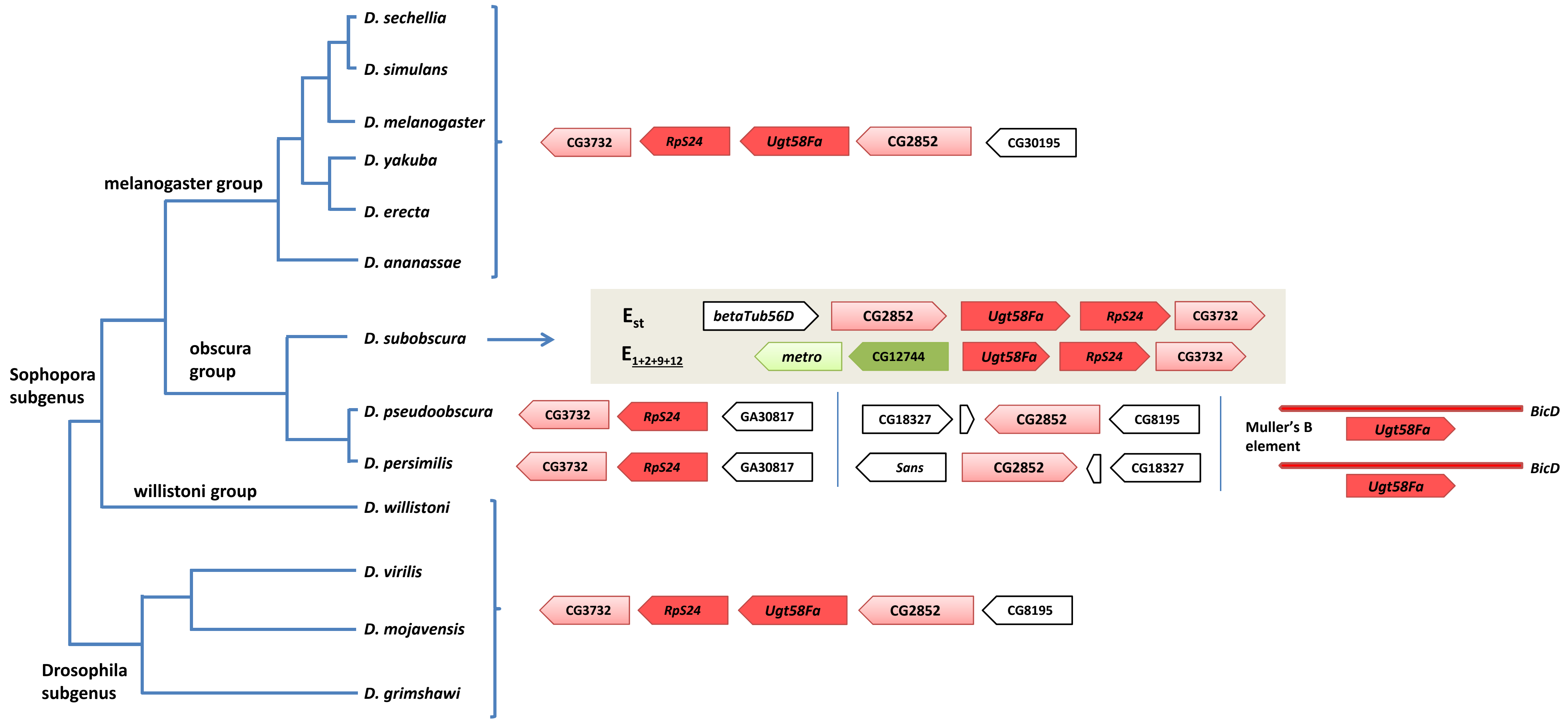

Supplementary Figure S6. Schematic representation of the genes flanking the CD breakpoint across the Drosophila phylogeny. Genes adjacent to the CD breakpoint and their neighbouring genes are represented by dark and light red boxes, respectively. Either green or clear boxes represent all other genes. All genes are named according to their *D. melanogaster* ortholog, except for gene GA30817 with a paralog in *D. pseudoobscura*. Genes are located in Muller's C element except gene *Ugt58Fa* that in *D. pseudoobscura* is located in Muller's B element within the span of gene *BicD* (represented by a thinner solid red box). In *D. subobscura*, the location of genes in both the E_{st} and $E_{1+2+9+12}$ arrangements is given.

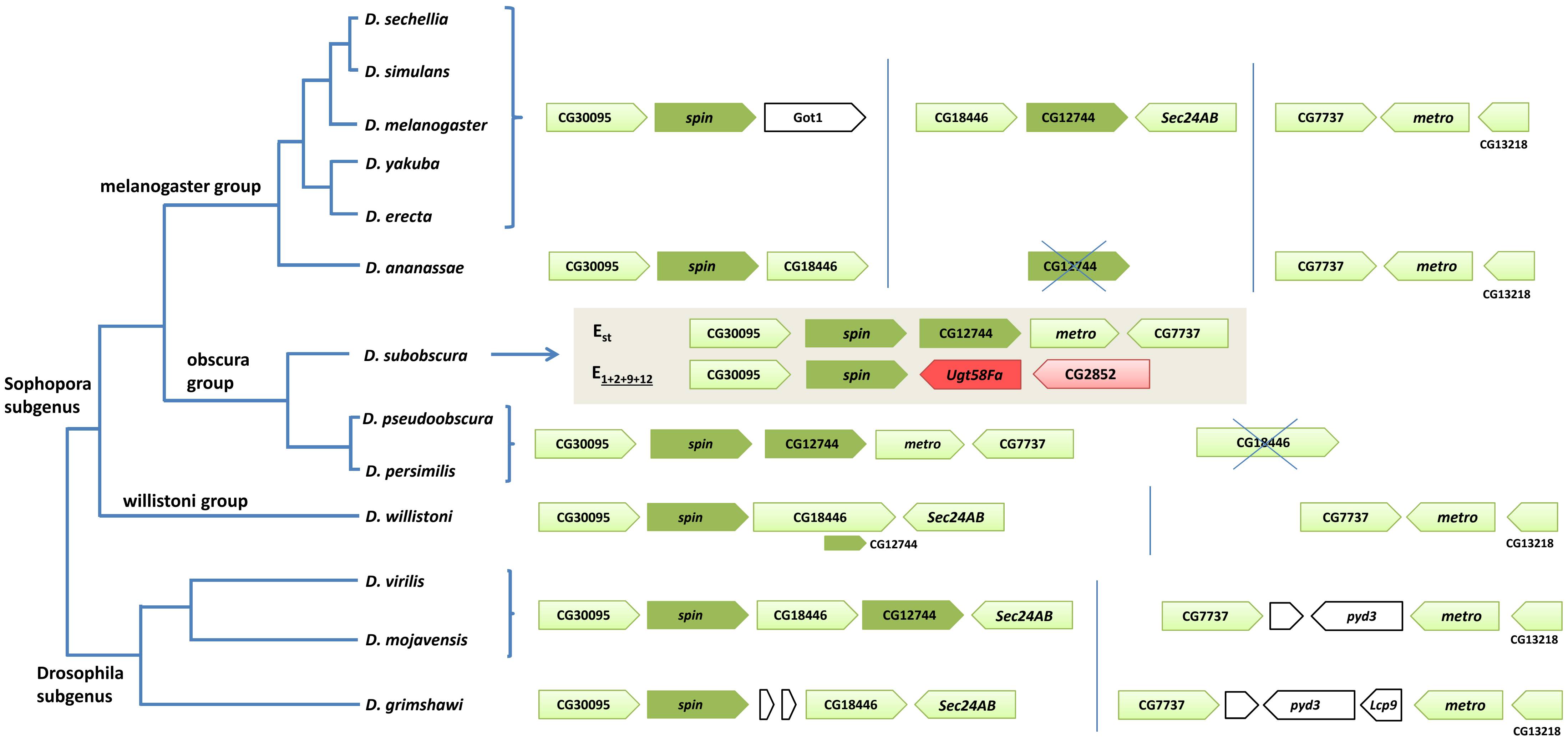

Supplementary Figure S7. Schematic representation of the genes flanking the IJ breakpoint across the Drosophila phylogeny. Genes adjacent to the IJ breakpoint and their conserved neighboring genes are represented by dark and light green boxes, respectively. Either red or clear boxes represent all other genes. All genes are named according to their *D. melanogaster* ortholog, except in those cases where no ortholog in *D. melanogaster* has been described. In the *D. subobscura* lineage, the location of genes in both the E_{st} and $E_{1+2+9+12}$ arrangements is given. Notice that the CG18446 gene is annotated in *D. melanogaster* within the span of gene *cbx* (not shown in the scheme).
